# Scabies: Application of the Novel Identify-Isolate-Inform Tool for Detection and Management

**DOI:** 10.5811/westjem.2020.1.46120

**Published:** 2020-02-21

**Authors:** Tabitha A. Cheng, Bandr Mzahim, Kristi L. Koenig, Abdulrahman Alsugair, Abdussalam Al-Wabel, Bandar Saad Almutairi, Eshmawi Maysa, Christopher A. Kahn

**Affiliations:** * University of California, San Diego, Department of Emergency Medicine, La Jolla, California; † King Fahad Medical City, Saudi Arabia; ‡ University of California, Irvine, Department of Emergency Medicine, Orange, California; § County of San Diego, Health & Human Services Agency, Emergency Medical Services, San Diego, California; ¶ Presidency of State Security, Emergency Consultant, Saudi Arabia

## Abstract

Scabies is a highly contagious, globally prevalent, parasitic skin infestation caused by *Sarcoptes scabiei* var. *hominis*, also known as the itch mite. There have been outbreaks not only in the developing world, but also in the developed world among refugees and asylum seekers. Once infested with scabies mites, symptomatic patients, as well as asymptomatic carriers, quickly spread the disease through direct skin-to-skin contact. Typically, symptoms of scabies are characterized by an erythematous, papular, pruritic rash associated with burrows. Treatment of scabies involves using topical or systemic scabicides and treating secondary bacterial infections, if present. Given the prevalence and contagiousness of scabies, measures to prevent its spread are essential. Through application of the novel Identify-Isolate-Inform (3I) Tool, emergency medical providers can readily identify risk factors for exposure and important symptoms of the disease, thus limiting its spread through prompt scabicide therapy; isolate the patient until after treatment; and inform local public health authorities and hospital infection prevention, when appropriate. Ultimately, these three actions can aid public health in controlling the transmission of scabies cases, thus ensuring the protection of the general public from this highly contagious skin infestation.

## INTRODUCTION

Human scabies is a highly contagious, globally prevalent, parasitic skin infestation caused by *Sarcoptes scabiei* var*. hominis*, also known as the itch mite. This parasite was identified in the 1687 by Bonomo and Cestoni using a light microscope; however, there is evidence of scabies as far back as 1200 BCE.^1^ The most common symptoms of scabies, itching and a skin rash, are caused by a hypersensitivity reaction to the proteins and feces of the parasite about four to six weeks after infestation. Severe pruritus, especially at night, is the earliest and most common symptom of scabies. An erythematous, papular, pruritic rash with burrows on the hands, wrists, torso, and feet is also common.^1^

Scabies continues to be a common dermatological disease internationally. A systematic review estimated the prevalence of scabies in various countries to be 0.2% to 71%.^2^ In the United Kingdom, a general practice database review for scabies estimated prevalence to be 2.2 and 2.8 per 1000 in men and women, respectively.^3^ Studies from Greece and Spain conducted in dermatology clinics concluded that scabies is encountered in approximately 4% of visits, particularly among immigrants and patients with low socioeconomic status.^4^,^5^ In developing countries the prevalence can be much higher,^6^–^9^ ranging as high as 87% in one study in Thai orphanages.^10^ Although prevalence of scabies is low in developed countries, public health authorities are challenged to identify and treat individuals with scabies promptly to avoid transmission amongst close-quartered populations, such as within the growing population of asylum seekers and refugees.^11^,^12^

Scabies remains a risk to public health, and it is essential that frontline healthcare providers identify potential cases. Both under- and over-diagnosis are possible, and each is problematic. While missing the diagnosis can lead to both ongoing individual patient discomfort as well as rapid population spread, over-diagnosis can lead to inappropriate individual patient treatment and can create stress on healthcare systems with finite resources.

**Figure 1:**
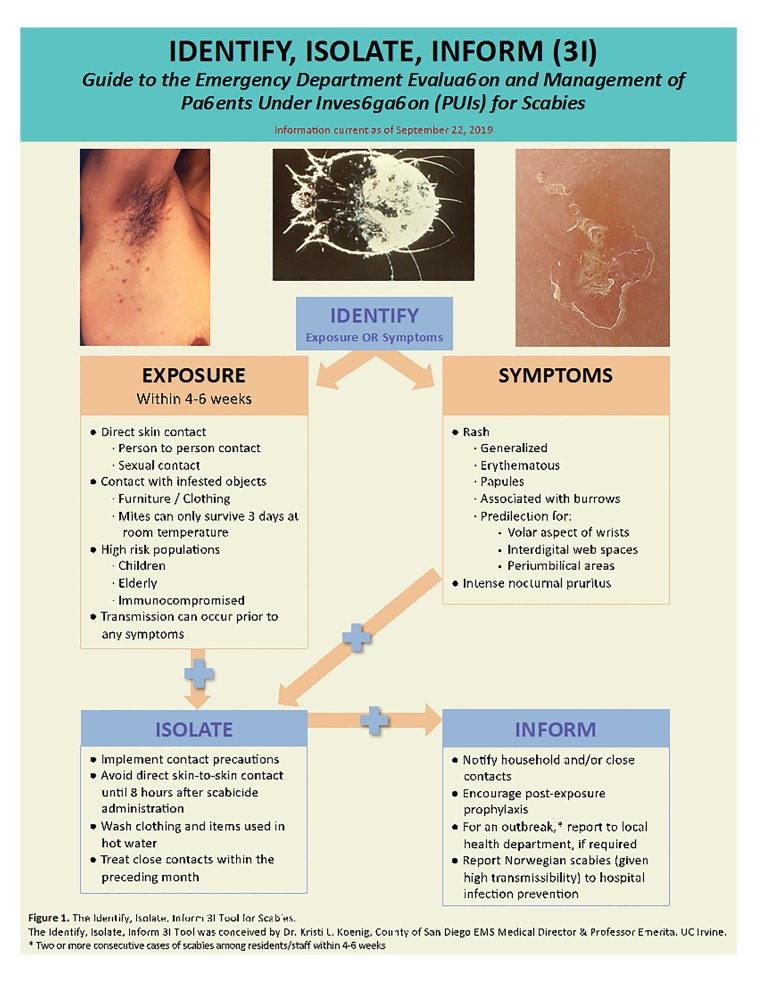
The Identify, Isolate, Inform (3I) Tool for Scabies.

Emergency Department (ED) providers may encounter and treat these patients as the first point of contact. After an overview of the disease and critical information pertaining to transmission and treatment, this article adapts the 3I (Identify-Isolate-Inform) Tool to assist frontline providers in the identification and management of potential cases of scabies presenting to the ED (Figure 1). The 3I Tool was originally developed for Ebola virus disease and subsequently modified for use in measles, Middle East Respiratory Syndrome (MERS), mumps, Zika, hepatitis A, pertussis, and 2019 nCoV (COVID-19).^13^–^20^

## CLINICAL PRESENTATION

Signs and symptoms of scabies differ depending on the time since exposure, degree of infestation, host immunocompetency, and coexistence of other skin pathologies. When people are first infested with scabies, they usually have no symptoms for 4–6 weeks. Classically, an intense nocturnal pruritic rash is the first symptom. The rash is typically characterized as erythematous with papules and associated burrows. Burrows are described as thin grey or brown lines that are approximately 5 mm long. Presence of burrows is a classic finding of scabies but uncommonly visualized due to skin excoriation or the presence of secondary infections. In adults and older children, the rash is most commonly found in the volar aspect of the wrists, interdigital web spaces, periumbilical area, anterior axillary folds, buttocks, and genitalia.^21^ In infants and those who live in tropical areas, the rash can be generalized and may also involve the scalp, neck, face, palms, and soles.^22^

## RISK FACTORS

Populations at highest risk for scabies include children, the elderly, the immunocompromised, and people in congregate living conditions, including refugee camps. Scabies is found worldwide and the risk of contracting infection is present regardless of gender, race, or socioeconomic status; however, higher prevalence of scabies has been correlated to tropical and subtropical climates, resource-poor countries, and areas with armed conflicts, homelessness, crowding, and shared use of clothes, beds, and blankets or pillows.^2^,^23^–^25^

## DIAGNOSIS

A presumptive diagnosis of scabies can be made based on suggestive clinical features such as nocturnal pruritus, history of contact with scabies, and/or typical appearance and distribution of skin lesions with the presence of burrows. However, achieving a definitive diagnosis depends on identification of mites, eggs, or fecal material using light microscopy. Lesions should be scraped off using a scalpel. With the scalpel, the papule should be scraped multiple times to remove the top ( Video Example: Scabies skin scraping technique ). Adding a few drops of mineral oil to the skin prior to scraping may help the scraped material to adhere to the blade. Most hospital pathology laboratories will accept scrapings for microscopic evaluations. The pathology protocols for scabies skin scraping methods at two university hospitals are provided here for reference: University of Iowa and University of Michigan. The characteristic microscopic appearance is shown here. Even with ideal technique, however, failure to find mites, eggs, or fecal material is common and does not rule out the disease.^26^,^27^ The sensitivity of this approach ranges from 40% to 90% and the specificity reaches 100%.^26^

**Population Health Research Capsule**

What do we already know about this issue?

Scabies is a highly contagious parasitic skin infestation with outbreaks in the developed world, as well as among at-risk populations in the developing world.

What was the research question?

Investigators modified the “Identify, Isolate, Inform” (3I) Tool for use in identifying and managing scabies.

What was the major finding of the study?

A novel Scabies 3I Tool is created for real-time application in emergency department (ED) patients.

How does this improve population health?

The Scabies 3I Tool aids ED providers who play an essential role in identifying and treating scabies effectively to avoid spread of the infestation.

**Figure 2:**
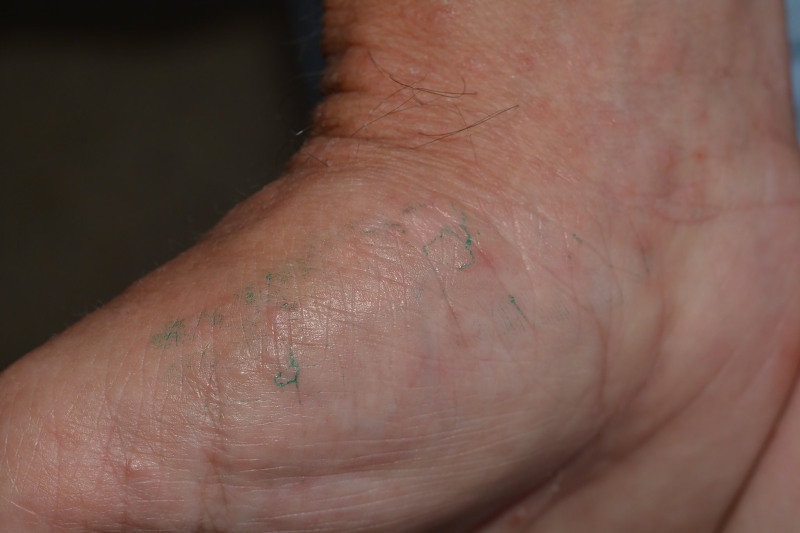
Scabies burrow ink test.^50^

Alternatively, the burrow ink test may be used. In this method, ink is absorbed by the burrows and will be visible as wavy lines (Figure 2).^24^,^25^ This method requires a dark felt tip washable marker or a fountain pen over the affected area and an alcohol swab to clean the surface ink. Any remaining dark ink under the skin signifies presence of scabies burrows (Figure 2). The sensitivity and specificity for the burrow ink test is unable to be calculated based on a French dermatology study.^26^ Nevertheless, for any case with concern for scabies in the emergency department, this is a simple test that may help diagnose scabies.

A substitute for the burrow ink test is the tetracycline fluorescence test, where tetracycline is used instead of ink.^28^,^29^ This method allows for colorless identification of the burrows. Similar to the ink, the topical tetracycline solution is applied over an affected area, and is wiped with alcohol to remove any excess solution on the surface. Then a Wood’s lamp is used to visualize the tetracycline that tracked into the burrows.^28^

Other scabies diagnostic techniques unlikely to be used in the emergency department include video dermatoscopy, polymerase chain reaction (PCR) and enzyme-linked immunosorbent assays (ELISA), and IgE antibody testing. Video dermatoscopy is especially useful in cases with atypical distribution or appearance of the lesions.^30^ Serological tests are emerging for both diagnosis and monitoring of treatment efficacy. One study showed that real-time PCR and ELISA tests are useful for monitoring treatment efficacy.^31^ Another study reported a 100% sensitivity and a specificity of 93% using IgE antibody against *Sarcoptes scabiei*.^32^

## COMPLICATIONS AND SPECIAL POPULATIONS

Secondary bacterial infections can develop in persons infested with scabies, particularly since the rash is typically intensely pruritic and scratching compromises the skin barrier and may introduce bacteria, particularly in patients with poor fingernail hygiene. Streptococcus or staphylococcus infections can cause impetigo, paronychia, cellulitis, or abscesses.^33^ Sequelae of these bacterial infections include bacteremia leading to sepsis, acute post-streptococcal glomerulonephritis, and rheumatic heart disease.^22^,^33^

The most vulnerable populations to scabies infestations are young children, the elderly, and the immunocompromised. These populations are especially susceptible to secondary complications of infestation. Given transmission is favored in conditions of crowding and poor sanitation, outbreaks have been seen in refugee camps and asylum seeker shelters.^12^

Crusted scabies, also known as Norwegian scabies, is particularly serious with a high mortality rate from bacterial sepsis.^34^ Caused by a hyperinfestation of the scabies mites, crusted scabies is characterized by development of a severe inflammatory response and hyperkeratosis (thickened skin crusts).^35^ Any skin area might be affected, but commonly affected regions include the scalp, hands, and feet. Crusts are malodorous and nails are thickened and discolored. Most cases of the crusted variant are linked to immunocompromised hosts; however, cases of crusted scabies have occurred without identifiable risk factors.^35^ From a public health perspective, patients with crusted scabies are highly infectious and, given they carry a significant number of mites, they can be the primary source of a community scabies outbreak. Furthermore, crusted scabies is difficult to manage, often requiring multiple treatments.^35^

## TRANSMISSION AND PERSONAL PROTECTIVE EQUIPMENT

Human scabies is a parasitic skin infection caused by penetration of the ectoparasitic mite *Sarcoptes scabiei* var. *hominis* into the epidermis. The lifecycle begins with a female mite laying eggs in the skin burrows. These larvae hatch, create new burrows, and then mature, mate and repeat this cycle.^1^ Once infested with scabies mites, symptomatic patients as well as asymptomatic carriers can easily spread the disease.^1^,^36^

Commonly, transmission occurs from person to person via direct skin contact, including by sexual contact. Because of the asymptomatic period following infestation, transmission can occur prior to symptom onset.^1^ In addition, fomite transmission through infested objects such as furniture and clothing is possible, especially with the crusted variant of scabies.^1^,^36^ Outside the human body and at room temperature with normal humidity, mites can only survive up to 3 days, whereas they are able to live up to 60 days inside human skin.^1^,^36^ Lower temperatures and higher humidity prolong survival of the mite off the host.^23^

To prevent transmission within healthcare facilities, patients should be in contact isolation until 8 hours after treatment.^36^ Personal protective equipment for healthcare workers treating patients with scabies includes the following: gowns, gloves, and shoe covers.^36^ Proper use of infection control measures including handwashing and avoiding skin-to-skin contact should also be used when handling patients with potential scabies infestations.

## DIFFERENTIAL DIAGNOSIS

The common manifestations of scaling and excoriation can impair skin visualization, making the differential diagnosis very broad. Clinicians should consider papular urticaria, secondary syphilis, folliculitis, contact dermatitis, atopic dermatitis, psoriasis, seborrhea, pityriasis rosea, lichen planus, and dermatitis herpetiformis as possible diagnoses.

## TREATMENT

Treatment options depend on whether scabies lesions are classic or crusted on clinical presentation (Table). In general, medications consist of a scabicide that can be applied topically or taken orally. For classic scabies, topical permethrin or oral ivermectin are considered first-line treatments.^23^ Dosing regimens are included in the table. Although high-quality trials comparing medications for the treatment of scabies are lacking, a Cochrane systematic review concluded that permethrin is more effective than ivermectin.^37^ A more recent 2018 systematic review, however, concluded that both ivermectin and permethrin have similar efficacy.^38^

When used as directed, topical permethrin 5% has high cure rates, approaching 90% in randomized trials.^39^ Permethrin is applied topically in patients older than 2 months of age from the neck to the soles of the feet and washed off after 8 to 14 hours. Considering that scabies can also affect the face, scalp and neck in infants and young children, topical application should be extended to these areas.^36^,^40^ Repeating the topical permethrin treatment one or two weeks after the first treatment is necessary in severe cases.^36^ Oral ivermectin is an alternate therapy that may be used if topical treatment fails; however, its safety in pregnant women and children weighing less than 15 kg has not been established.^34^,^46^,^37^,^41^–^43^

For crusted scabies, both an oral and a topical scabicide should be administered concurrently (Table).^15^,^19^,^37^ Ivermectin use has also been described for scabies control in endemic areas or outbreaks where topical scabicide use may be difficult.^12^ A randomized controlled trial examining mass administration of ivermectin for management of scabies concluded a reduction in prevalence from 32.1% to 1.9% in the ivermectin group compared to a reduction from 41.7% to 15.8% in permethrin-treated controls.^44^

Other topical agents such as sulfur, benzyl benzoate, crotamiton, and lindane are also options if first-line treatments fail. Topical sulfur is considered safe when used to treat infants younger than 2 months of age and pregnant women.^36^,^40^,^41^ In addition to scabicides, treatment of secondary bacterial infections such as pyoderma or impetigo, if present, is indicated via administration of appropriate systemic antibiotics.^36^ Advising patients and parents of young patients to keep fingernails short and clean can assist with preventing secondary infections.

Patients treated for scabies may have persistent pruritus for up to 4 weeks. Many patients return to the ED with concerns of treatment failure or reinfestation. These persistent symptoms do not necessarily indicate treatment failure. Symptomatic treatment and reassurance are often the only necessary management. Symptoms that persist or worsen beyond 2 to 4 weeks, especially if the rash worsens or new burrows appear, should trigger the physician to consider other causes such as incorrect diagnosis, treatment failure due to resistance or incorrect application, secondary infections, and/or reinfestation.^45^

## PREVENTION

To eradicate and prevent reinfestation of the scabies mites, close contacts (within the previous 30 days) should be treated simultaneously. Additionally, items used by patients and close contacts in the preceding several days such as clothing and linens should be washed and dried at high temperatures (≥60°C), dry-cleaned, or placed in a plastic bag for at least 72 hours if unable to launder.^36^,^46^ Another aspect of controlling this disease is avoidance of direct skin-to-skin contact with suspected or confirmed cases of scabies until 8 hours after treatment.^36^ Treatment failures occur in some instances due to improper or inadequate application of the medications, reinfestation secondary to mishandling of clothes and bed linens, undertreatment of close contacts, and resistance to some medications, such as lindane.^34^,^35^,^37^,^41^–^43^,^47^ Prevention and control of crusted (Norwegian) scabies is more complicated given that brief skin-to-skin contact can spread the infection. In these cases, numerous contacts may need treatment to prevent a large-scale outbreak.^35^,^41^ In communities with a high prevalence of scabies, mass drug administration of scabicides has been used for effective control. This may be a strategy for large outbreaks; however, local health authorities should be consulted prior to instituting this approach.^48^

## DISPOSITION

Hospitalization is not recommended in patients with scabies unless they have other indications, such as crusted (Norwegian) scabies or severe secondary infections. Follow-up care 2–4 weeks after treatment should be arranged to assess for medication failure as well as reinfestation.^38^ Based on suggested general guidelines by the Centers for Disease Control and Prevention, patients with scabies may return to work or school 1 day after starting treatment and prior to follow-up.^36^ Healthcare providers with scabies who deliver direct hands-on care to patients and remain symptomatic after beginning treatment may return to work if they observe standard precautions, including the use of disposable gloves, until they are sure they are no longer infested.^36^

## IDENTIFY-ISOLATE-INFORM (3I)

The Identify-Isolate-Inform (3I) Tool was conceived during the 2014 Ebola virus disease outbreak and later modified for application to the ED evaluation and management of patients with other communicable diseases.^14^–^20^ The novel modification of this tool presented here can be applied for ED evaluation and management of a patient under investigation for scabies. The Scabies 3I Tool is an algorithm that begins with *identifying* suspected cases based on symptoms, exposure history, and testing as needed. History of exposure is important as patients can transmit scabies prior to symptom onset. Identification of close contacts is also an important step in controlling the spread of the infestation.

To prevent transmission within healthcare facilities, patients should be *isolated* in contact isolation until 8 hours after treatment.^36^ Personal protective equipment for healthcare workers treating patients with scabies includes the following protective garments: gowns, gloves, and shoe covers.^36^ Proper use of infection control measures including avoiding skin-to-skin contact and handwashing should be observed when handling patients with potential scabies infestations.^36^

Given the transmissibility of this disease and potential outbreaks that may threaten public health, ED staff should immediately *inform* the local health authority in cases of outbreak, defined as two or more consecutive cases of scabies among residents/staff within 4–6 weeks.^49^ Timely notification of an outbreak is especially important in cases of scabies identified from healthcare facilities, shelters, or other communities where the disease could rapidly spread, including refugee and migrant shelters. In an online review of 20 hospital policies across the United States, no hospital required informing hospital infection control of scabies cases; however, individual hospital policies may vary both within the U.S. and internationally. Therefore, it is important to know and follow local hospital policies on scabies reporting. Additionally, cases of crusted (Norwegian) scabies should be isolated promptly and all close contacts should be informed and treated, given its high transmission rate.^35^,^41^ Using this 3I Identify-Isolate-Inform Tool, healthcare providers can be more prepared to detect and manage potential scabies cases.

## CONCLUSION

Prompt recognition of transmittable diseases, like scabies, by emergency healthcare workers is needed to mitigate spread. Scabies can be challenging to diagnose, and both under- and over-diagnosis of scabies have negative health and resource consequences. The novel Scabies Identify-Isolate-Inform (3I) Tool can aid ED staff in readily recognizing key risk factors for exposure and characteristic symptoms of the disease, thereby triggering implementation of appropriate isolation protocols, and notification of hospital and public health agencies, as appropriate.

## Figures and Tables

**Table:** Medication dosing and timing regimens for scabies with variation for Norwegian (crusted) scabies.

Medication	Dosing	Timing	Variation for Norwegian scabies
Permethrin cream 5%	Apply topically entire body	Wash off after 8–14 hours	Repeated daily for 7 days then twice weekly until discharge or cure* *Recommended combination therapy with ivermectin
Ivermectin	200 mcg/kg/dose orally	2 doses 1 week apart	3, 5 or 7 doses depending on severity 3 dose regimen: days 1, 2, 8 5 dose regimen: days 1, 2, 8, 9, 15^#^ 7 dose regimen: days 1, 2, 8, 9, 15, 22, 29 *^#^Recommended combination therapy with permethrin*
Sulfur ointment (5%–10%)	Apply topically entire body	Wash off after 24 hours Repeat for 3 doses	Not recommended

*Mcg/kg/dose*, micrograms per kilogram per dose.
